# Misestimation of heritability and prediction accuracy of male-pattern baldness

**DOI:** 10.1038/s41467-018-04807-3

**Published:** 2018-06-29

**Authors:** Chloe X. Yap, Julia Sidorenko, Riccardo E. Marioni, Loic Yengo, Naomi R. Wray, Peter M. Visscher

**Affiliations:** 1 Institute for Molecular Bioscience, The University of Queensland, Brisbane, 4072 Australia; 2 Estonian Genome Center, Institute of Genomics, University of Tartu, Tartu, 51010 Estonia; 3 Centre for Genomic and Experimental Medicine, Institute of Genetics and Molecular Medicine, University of Edinburgh, Edinburgh, EH4 2XU UK; 4 Centre for Cognitive Ageing and Cognitive Epidemiology, University of Edinburgh, Edinburgh, EH8 9JZ UK; 5 Queensland Brain Institute, The University of Queensland, Brisbane, 4072 Australia

Pirastu et al.^1^ perform the largest GWAS to date on male-pattern baldness (MPB), discover 71 loci (of which 30 are new) and draw inference about its heritability and genetic architecture. They report a SNP heritability on the scale of liability (*h*_*l*_^2^) of 94%, with 38% of total heritability explained by the 71 loci. From these estimates, they draw strong conclusions about the genetic architecture of MPB. However, the chosen definition of the phenotype and the applied transformation to the unobserved scale of liability have led to a large upwards bias of the estimates of these parameters, as shown here in theory and from data.

In the UK Biobank (UKB), MPB is measured on a four-point ordinal scale (values 1–4, with 1 representing no sign of baldness). Using the same UKB sub-sample selection as Pirastu et al. (unrelated British, genetically Caucasian, *n* = 54,813), the proportion of men with self-report MPB in each category is 0.317, 0.229, 0.269 and 0.185, respectively. In analysis, the authors ignore 23% of the population with a score of 2, and define ‘cases’ as those with self-reported scores of 3 or 4, and ‘controls’ as self-reported scores of 1, leading to a ‘prevalence’ of 59%. Yet the reported *h*_*l*_^2^ estimates are presented as if parameters in the (whole) population. An implicit assumption of their approach is that those self-reporting a score of 2, which they consider to be ‘rather dubious baldness’, are randomly drawn from the population. To determine if this assumption is valid, we took the 47 most associated independent autosomal loci that were identified independently^2–6,10^ of the UKB data (to avoid bias) and then used the same UKB data as in Pirastu et al. to estimate the frequencies of the trait-increasing alleles for each of the 4 scores. The results (Fig. 1) show that these frequencies are approximately linear in scores 1–4, and clearly score 2 is not random with respect to liability. Moreover, the observed pattern is consistent with an additive model on the scale of these scores. Therefore, since a score of 2 is correlated with liability to MPB, ignoring individuals with a score of 2, without accounting for the resulting extreme tail ascertainment, will lead to a bias in the estimate of genetic parameters. We derived from theory the general transformation equation that should be applied to the estimate of heritability made on the binary observed scale in samples that are ascertained based on tail selection and/or oversampling of cases or controls ($h_{o[s]}^2$) to achieve unbiased estimates of *h*_*l*_^2^ (equation [1] in Supplementary Methods).

**Fig. 1:**
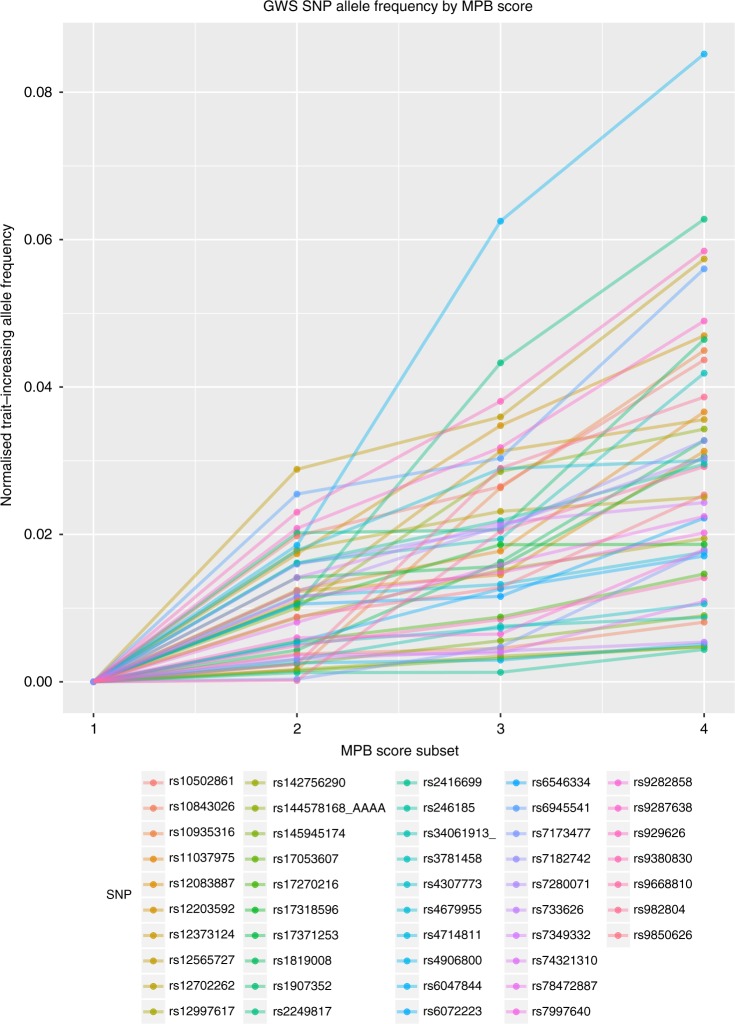
Trait-increasing allele frequency by MPB score in UKB for 47 genome-wide significant GWAS loci identified in refs. ^2–6,10^. For each of the 47 loci, the trait-increasing allele frequency in the UK Biobank sample is given on the *y*-axis, as a deviation from its frequency for men with a MPB score of 1. The *x*-axis labels represent the observed MPB categories in the UK Biobank

We first replicated the results of Pirastu et al., using their sampling design and model (as best as we could deduce from the details provided) and using the same UK Biobank data. The estimate $h_{o[s]}^2$ for scores 3 + 4 vs. score 1 using GCTA^7^ was 0.61 (s.e. = 0.03). If this is transformed to the scale of liability using the standard equation^8^ (equation [2] in Supplementary Methods) then the estimate of *h*_*l*_^2^ is 0.98 (standard error, s.e. = 0.04) similar to the estimate reported by Pirastu et al. However, the correct transformation (equation [1] in Supplementary Methods) generates an estimate of 0.64 (s.e. = 0.03). To empirically explore assumptions of the liability threshold model, we analysed random samples of 20,000 males dichotomised in a number of ways (Table 1). These analyses generated estimates of *h*_*l*_^2^ in the range of 0.61–0.75. We also analysed MPB on the continuous scale of 1–4, which does not remove information through dichotomisation, transforming the estimate of heritability to the liability scale *h*_*l*_^2^ = 0.69 (s.e. = 0.03)^9^ (equation [3] in Supplementary Methods).

**Table 1:** Estimates of heritability of liability of MPB using different random samples of 20,000 men ascertained in different ways

MPB scores for cases	MPB scores for controls	$K_{\mathrm{L}}$	$K_{\mathrm{U}}$	*P*	$h_{o[s]}^2({\mathrm{s}}.{\mathrm{e}}.)$	$h_l^2({\mathrm{s}}.{\mathrm{e}}.)$	*R* ^*2*a^
4	1,2,3	0.81	0.19	0.19	0.36 (0.03)	0.75 (0.06)	0.15
3,4	1,2	0.54	0.46	0.46	0.46 (0.03)	0.72 (0.05)	0.16
2,3,4	1	0.68	0.32	0.32	0.41 (0.03)	0.70 (0.05)	0.17
3,4^b^	1	0.32	0.46	0.59	0.61 (0.03)	0.64 (0.03)	0.16
4	1	0.32	0.19	0.37	0.96 (0.03)	0.63 (0.02)	0.13
Quantitative 1,2,3,4					0.59 (0.03)	0.69 (0.03)	0.16

*K*_L_ proportion of the population in the lower tail, extreme controls. *K*_U_ proportion of the population in the upper tail, cases. *P* proportion of the samples used for analyses that are cases.

^a^Proportion of variance in liability explained by the 107-SNP predictor

^b^The sampling strategy conducted by Pirastu et al.

We estimated the variance explained by the 107 SNP predictor from the difference in the estimate of total phenotypic variance in models excluding and including the predictor as a fixed effect. This method for estimation of the contribution of the SNP predictor to trait variation differs to that presented by Pirastu et al. In contrast to their approach, it does not depend on unbiased estimation of genetic variance in the two models. Moreover, it is accurate (the s.e. of estimating a phenotypic variance is small) and quantifies a parameter that is most relevant to epidemiology and risk prediction. From the estimate of the variance explained by the predictor, we calculated the proportion of variance it explained on the observed scale and then transformed this proportion to the scale of liability. Results (Table 1) imply that the variance in liability attributable to this predictor is ~15–20%, substantially less than claimed by the authors.

In conclusion, the evidence presented by Pirastu et al. is not consistent with the claims that virtually all variation in liability to MPB is genetic and that common SNPs capture all that variation. A correct transformation from the observed scale to a scale of liability results in an estimate of SNP heritability of ~60–70%, and the 71-loci (107-SNP predictor) explains about 15–20% of variation in liability.

## Electronic supplementary material

Supplementary Information
