# Author Correction: Misestimation of heritability and prediction accuracy of male-pattern baldness

**DOI:** 10.1038/s41467-018-07400-w

**Published:** 2018-11-20

**Authors:** Chloe X. Yap, Julia Sidorenko, Riccardo E. Marioni, Loic Yengo, Naomi R. Wray, Peter M. Visscher

**Affiliations:** 1 Institute for Molecular Bioscience, The University of Queensland, Brisbane, 4072 Australia; 2 Estonian Genome Center, Institute of Genomics, University of Tartu, Tartu, 51010 Estonia; 3 Centre for Genomic and Experimental Medicine, Institute of Genetics and Molecular Medicine, University of Edinburgh, Edinburgh, EH4 2XU UK; 4 Centre for Cognitive Ageing and Cognitive Epidemiology, University of Edinburgh, Edinburgh, EH8 9JZ UK; 5 Queensland Brain Institute, The University of Queensland, Brisbane, 4072 Australia

Correction to: *Nature Communications*; 10.1038/s41467-018-04807-3; published online 29 June 2018

The original version of this Article contained an error in the spelling of the author Julia Sidorenko, which was incorrectly given as Julia Sirodenko. This has now been corrected in both the PDF and HTML versions of the Article. Further, the sixth sentence of the second paragraph of the Correspondence and the legend to Fig. 1 incorrectly omitted citation of work by Heilmann-Helmbach, S. et al. This has now been corrected in both the PDF and HTML versions of the Article.

Heilmann-Helmbach, S. et al. Meta-analysis identifies novel risk loci and yields systematic insights into the biology of male-pattern baldness. *Nat. Commun.* **8**, 14694 (2017).

