# Supplementary material for: Misestimation of heritability and prediction accuracy of male-pattern baldness
Source: Nat Commun. 2018 Jun 29;9:2537. doi: 10.1038/s41467-018-04807-3 (PMC6026149; doi:10.1038/s41467-018-04807-3)
Supplement: Supplementary file 1 — Supplementary Information [file 41467_2018_4807_MOESM1_ESM.pdf]

Supplementary Information

Correspondence: Mis-estimation of heritability and prediction accuracy of male-pattern baldness

Yap et al.

Supplementary Methods

Oversampling of cases and/or controls

We assume a standard liability model $y_l = g_l + e_l$, with var(y_l) = 1, var(g_l) = h_l^2 and var(e_L) = 1-h_l^2. Here, y_l is on the unobserved scale of liability and h_l^2 is its heritability. We assume an upper K_U proportion of the population are cases, the lower K_L proportion of the population are extreme controls, and that in an ascertained sample the proportion of cases is P and the proportion of controls is (1-P). The corresponding heights of the normal curves pertaining to proportions K_U and K_L are z_L and z_U, respectively.

Lee et al. (2011)[1] and Golan et al. (2014)[2] derive the expected heritability on the observed 0-1 scale estimated from a sample of cases and controls ($h_{o[S]}^2$) for the case of $K_L = 1 - K_U$. Their results can be expressed as,

$$E[h_{o[S]}^2] = h_l^2 P(1-P)(\frac{z_U}{K_U} + \frac{z_U}{(1-K_U)})^2$$

It follows from the derivations in Golan *et al.* (2014)[2] that this equation generalises to the approximation for the relationship between heritability on the observed scale and the scale of liability when cases and/or controls are over-sampled from the population. In particular, for arbitrary values of K_U and K_L (and subsequently different values of z_L and z_U),

$$E[h_{o[S]}^2] = h_l^2 P(1-P)(\frac{z_U}{K_U} + \frac{z_L}{K_L})^2$$

Therefore, the estimated value $h_{o[S]}^2$ can be transformed to the liability scale as,

$$h_l^2 = \frac{h_{o[S]}^2}{P(1-P)(\frac{z_U}{K_U}+\frac{z_L}{K_L})^2} \qquad [1]$$

When $K_L = 1 - K_U$ as in[1,2], then $z_L = z_U$ and the transformation simplifies as

$$h_l^2 = \frac{h_{o[S]}^2(K_U(1-K_U))^2}{P(1-P)z_U^2}. \qquad [2]$$

Analysis on the observed 1-4 scale

We assume a multiple threshold model underlying the observed 1-4 scores. Gianola (1979)[3] derived $h_{o[o]}^2$ for any linear combination of scores in n ordinal categories. In the case of observations on MPB, where n=4 and Y_0 takes on values 1-4 with frequencies π_j,

$$E[h_{o[o]}^2] = \frac{h_l^2(\sum_{j=1}^{n-1} z_j)^2}{\mathrm{V}(Y_o)}$$

with z_j the height of the normal curve corresponding to threshold j (there are 4 categories so 3 thresholds), and V(Y_o) = $\sum_{j=1}^{n} \pi_j w_j^2 - (\sum_{j=1}^{n} \pi_j w_j)^2$, with w_j taking on values 1, 2, 3 and 4. Therefore,

$$h_l^2 = \frac{h_{o[o]}^2 V(Y_o)}{(z_1+z_2+z_3)^2} \qquad [3].$$

Supplementary References

1. Lee, S.H., Wray, N.R., Goddard, M.E. & Visscher, P.M. Estimating Missing Heritability for Disease from Genome-wide Association Studies. *Am J Hum Genet* **88**, 294-305 (2011).
2. Golan, D., Lander, E.S. & Rosset, S. Measuring missing heritability: inferring the contribution of common variants. *Proc Natl Acad Sci U S A* **111**, E5272-81 (2014).
3. Gianola, D. Heritability of polychotomous characters. *Genetics* **93**, 1051-5 (1979).
